# Supplementary material for: Kernel-based gene–environment interaction tests for rare variants with multiple quantitative phenotypes
Source: PLoS One. 2022 Oct 12;17(10):e0275929. doi: 10.1371/journal.pone.0275929 (PMC9555665; doi:10.1371/journal.pone.0275929)
Supplement: Attachment — Submitted filename: Response to Reviewers.doc [file pone.0275929.s001.doc]

**Responses to the Reviewer:**

1. Have you considered comparing your four proposed methods with current existing methods in GEI pleiotropy field via simulations? Also, can you discuss what are the advantages of the proposed models and limitations of the existing methods?

**Response**: Thank you for the suggestion! We conducted an extensive literature search and found only one method available for testing GEI with rare variants and multiple phenotypes (Zhang et al. 2019). The method consists of three steps: remove correlation among multiple phenotypes by using principal component analysis or other linear transformations; obtain p value for each transformed phenotype by testing the effects of an optimally weighted combination of gene-environment interactions for rare variants (TOW-GE) (Zhao et al. 2020); employ Fisher’s combination test (FCT) to combine the p values of multiple phenotypes. We denote the method as TOWGE-FCT in this paper. It can be expected that the degree of freedom of TOWGE-FCT would become larger with the increasing number of phenotypes, which might limit statistical power of the test.

In the revised manuscript, we have added simulations and compared our proposed four multiphenotype GEI tests with TOWGE-FCT. The results showed that all the five tests have enhanced power as the number of phenotypes associated with interactions increases, our proposed Het-GEI, PPK-GEI and LPK-GEI had larger power than TOWGE-FCT, but Hom-GEI had smaller power than TOWGE-FCT. Moreover, TOWGE-FCT uses a permutation test to estimate p value for each phenotype, which is very time consuming. In comparison, our proposed four tests have asymptotic distributions and p values can be computed analytically. Thus, our proposed tests have much smaller computational cost. We also added discussion on the advantages of our proposed tests and limitations of TOWGE-FCT in the revised manuscript.

Zhang J, Sha Q, Hao H, Zhang S, Gao XR, Wang X. Test Gene-Environment Interactions for Multiple Traits in Sequencing Association Studies. Hum Hered. 2019;84(4-5):170-196. <https://doi.org/10.1159/000506008> PMID: 32417835

Zhao Z, Zhang J, Sha Q, Hao H. Testing gene-environment interactions for rare and/or common variants in sequencing association studies. PLoS One. 2020;15(3):e0229217. <https://doi.org/10.1371/journal.pone.0229217> PMID: 32155162

2. In the UK Biobank data application, there are several genes identified by your proposed models to have interactions. Are these genes confirmed in past literature to show correlations with BP phenotypes? Have you tried anyway to validate the results as well as your models?

**Response**: Thank you for the suggestion! In our work, we identified three genes that have interactions with Hb in BP phenotypes. For *NOS3* and *MYO1C* genes, they have been reported to be correlated with BP phenotypes in (Surendran et al. 2020).

For *LEUTX* gene, in an analysis of 454,787 UK Biobank participants, *LEUTX* was not associated with the BP phenotypes at the nominal significance level of 0.05 (Backman et al. 2021). While our proposed tests identified *LEUTX* that shows interaction with Hb in BP phenotypes, the result lacks independent validation. Thus, it should be considered as being preliminary and further experiments are necessary. We acknowledged the limitation of the result in the revised manuscript.

Surendran P, Feofanova EV, Lahrouchi N, Ntalla I, Karthikeyan S, Cook J, et al. Discovery of rare variants associated with blood pressure regulation through meta-analysis of 1.3 million individuals. Nat Genet. 2020;52(12):1314-1332. <https://doi.org/10.1038/s41588-020-00713-x> PMID: 33230300

Backman JD, Li AH, Marcketta A, Sun D, Mbatchou J, Kessler MD, et al. Exome sequencing and analysis of 454,787 UK Biobank participants. Nature. 2021;599(7886):628-634. <https://doi.org/10.1038/s41586-021-04103-z> PMID: 34662886
